# Development of Series of Affinity Tags in *Streptomyces*

**DOI:** 10.1038/s41598-017-07377-4

**Published:** 2017-07-31

**Authors:** Xu-Ming Mao, Ning Sun, Yang Zheng, Yong-Quan Li

**Affiliations:** 1 Institute of Pharmaceutical Biotechnology, College of Pharmaceutical Sciences, Zhejiang University, Hangzhou, 310058 China; 2 Zhejiang Provincial Key Laboratory for Microbial Biochemistry and Metabolic Engineering, Hangzhou, 310058 China; 3 Present Address: Department of Biological Sciences, Duquesne University, Pittsburgh, Pennsylvania 15282 USA

## Abstract

*Streptomyces* are of great biological and industrial significance due to their complex morphological development and ability to produce numerous secondary metabolites. However, the intrinsic biochemical mechanisms underlying morphogenesis and secondary metabolism are rarely revealed, partially because of the limited availability of the biochemical tools in *Streptomyces*. Here we provided series of integrative vectors with various affinity tags, including single tags 3×FLAG, 3×HA, 3×Strep-tag II, 18×His, 13×Myc, and dual tags, all of which were driven from a strong constitutive promoter ermEp*. Using a sigma factor SigT from *S. coelicolor* as a model, we successfully expressed and immuno-detected SigT fused with all tags. Moreover, after SigT was N-terminally tagged with 3×FLAG and C-terminally tagged with 18×His, we isolated SigT-interactive proteins from the *S. coelicolor* lysate based on the tandem affinity purification (TAP). Particularly, among the proteins purified, the SigT cognate anti-sigma factor RstA ranked the top with the most total independent spectra. These data suggested the feasibility of these affinity tags in *Streptomyces*, which will be widely employed to explore the biochemical mechanisms to further understand the dynamic and elaborate regulation in this genus.

## Introduction

*Streptomyces*, the soil-dwelling filamentous bacteria, possess a periodic morphological development with a progression of cell types from vegetative mycelia to aerial mycelia, and to the production of spores, and have a complex secondary metabolism to produce invaluable antibiotics, immuno-suppressors, anti-tumor drugs, etc^1^. Due to their biological, industrial and clinical importance, numerous efforts have been made to reveal the regulatory pathways and their cross-talks, mainly at the genetic and transcriptional levels, all of which will be the basis to engineer the bacteria in this genus by systems biology, synthetic biology and metabolic engineering, etc^2–7^.

Abundant genetic tools, including transposons, linear/circular and high/low-copy vectors, have been developed initially to elucidate the gene functions and genomic information of *Streptomyces* ^8^. Complicated regulatory mechanisms have also been dissected based on the well-developed biochemical assays, such as *in vitro* transcription, electrophoretic mobility shift assay (EMSA) combined with *in vivo* assays, such as chromatin immunoprecipitation (ChIP). Particularly, the *bld* genes, *whi* genes, and several sigma factors essential for aerial hyphae development and sporulation have been discovered and the underlying genetic circuits were depicted^9, 10^. The highly conserved signaling pathways triggered by γ-butyrolactones but mediated by the pleiotropic regulator AdpA have been shown to globally regulate morphological transition and secondary metabolism^9, 11, 12^. Moreover, the nutrient-sensing pathways, such as the PhoRP two-component system and the orphan regulator GlnR, are also found in regulation of secondary metabolite production independently or in cross-talks^13–16^.

However, the regulatory complexity of *Streptomyces*, which essentially results from the *in vivo* dynamic protein turn-over, transient participation of particular proteins in different complexes, and competition or coordination of multiple regulatory factors on the regulons in a developmental phase-dependent manner, has been scarcely reported to date. Exploration of these mechanisms raises the requirement to develop protein-specific antibodies, or alternatively to label proteins with small tags without interference with their authentic functions. Along with commercial availability of high quality antibodies, several small affinity tags have been extensively used, especially in eukaryotic cells, to study the dynamic protein-protein and protein-DNA interaction, map the interactome, and reveal the protein complexes in different organelles, which has significantly helped us to understand their biology in nature and related diseases, and dramatically saved time and labors simultaneously^17–19^.

In *Streptomyces*, limited tags have been successfully used, such as FLAG in ChIP assays^10, 20^, eGFP for protein degradation monitoring^21–23^, and both tags for protein-protein interaction assays^22^. eGFP and mCherry have tremendously facilitated visualization of dynamic localization of morphogenesis-related proteins, revealing the subtle morphological changes and authentic protein functions^24–26^. However, these bulky fluorescent proteins might cause steric hindrance to impede the appropriate protein folding^27^ and block the interaction with others^28^.

Here we provided series of *Streptomyces* vectors containing various codon-optimized small single tag or dual tags, including 3×FLAG, 3×HA, 13×c-Myc, 18×His and 3×Strep-tag II. All these tags have been successfully expressed in fusion with the sigma factor SigT in *S. coelicolor*, and tandem affinity purification (TAP) was demonstrated for the first time in *Streptomyces* to show the feasibility of these tags without functional disruption of the fused proteins.

## Results

### Construction of *Streptomyces* vectors with affinity tags

Antibodies are powerful tools in investigating protein functions nowadays. Both protein-specific polyclonal and monoclonal antibodies against endogenously expressed PhoP and ScbR2 have been reported in *Streptomyces* ChIP assays^16, 29^. However, though polyclonal antibodies can be readily raised, quality control for affinity and specificity is problematic, and preparation of monoclonal antibodies will be time-consuming and risky^30, 31^. One of resolutions to overcome these problems is to express the target proteins in fusion with commonly used small epitopes, such as 6×Histidine, 8-amino acid FLAG tag (DYKDDDDK), 9-amino acid influenza hemagglutinin (HA) tag (YPYDVPDYA), 10-amino acid c-Myc tag (EQKLISEEDL), etc^32^. The 8-amino acid Strep-tag II (WSHPQFEK) is a recently developed epitope exhibiting intrinsic affinity toward streptavidin, and can be used to efficiently purify fusion proteins by Strep-Tactin conjugated beads^33^. Fusion of one of these small tags to the target proteins has been proven to have the minimal influence on their native conformation and functions^34^. Moreover, there are commercially available monoclonal antibodies against these affinity tags for various purposes, such as immunoblot, immunoprecipitation (IP), immunohistochemistry (IHC) and ChIP, with the advantages of predominantly high affinity, specificity, and time- and labor-saving.

**Figure 1:**
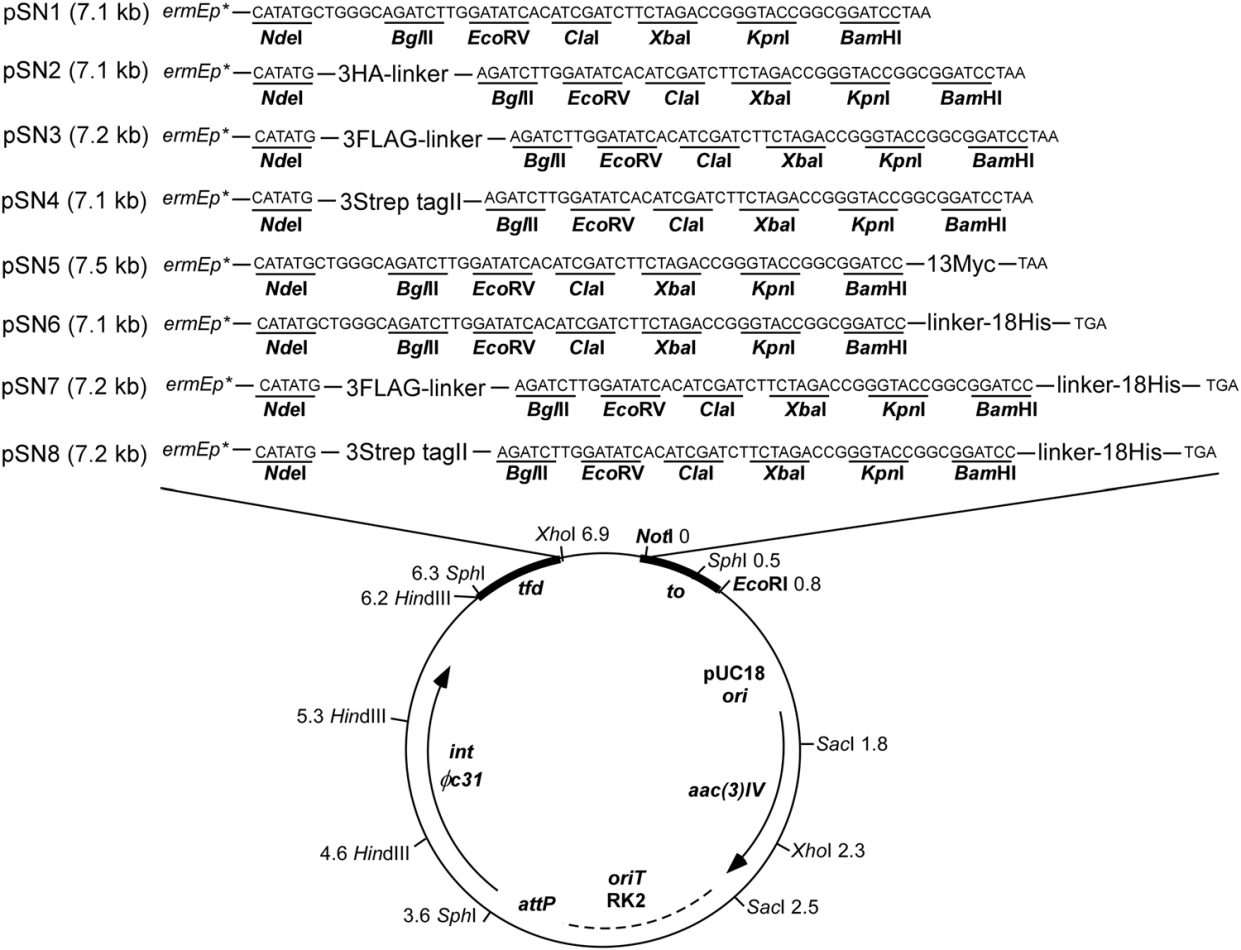
Maps of series of vectors pSN1-pSN8 with affinity tags. These vectors were the derivatives of pIJ8660. All the expression cassettes were driven from a strong promoter ermEp*, and multiple cloning sites (MCS) were shown in bold.

To expand their applications and increase their antigenicity in *Streptomyces*, multiple tandems of these small epitopes were generated after codon-optimization. The 22-amino acid 3×FLAG (DYKDHDGDYKDHDIDYKDDDDK), and tandemly arrayed small tags including 3×HA, 13×Myc, 18×His, 3× Strep-tag II were all expressed under the control of a strong constitutive promoter ermEp*^22^. Eight vectors (pSN1-pSN8) (Fig. 1) were the derives of the integrative promoter-probing vehicle pIJ8660^35^, where ermEp* was inserted between two *Bgl*II sites to remove the multiple cloning sites (MCS) on the parental vector, and the *egfp* reporter gene was replaced by the synthetic DNA fragments encoding the tandem epitopes together with the redesigned MCS. To meet the demand of labeling proteins at alternative ends, the 18×His tag was positioned at the C-terminus of the fusion proteins, while others were N-terminally tagged. Meanwhile, for the purpose of tandem affinity purification (TAP), two vectors were constructed by combination of N-terminal 3×FLAG or 3× Strep-tag II with C-terminal 18×His, given that 18×His-tagged fusion proteins can be purified both at native and denatured conditions, and the purification matrix Ni^2+^-NTA is relatively inexpensive, while anti-FLAG beads and Strep-Tactin beads are in excellent quality control in protein purification and immunoprecipitation^10, 33^. Moreover, to avoid possible steric hindrance from the tandem epitopes, a linker sequence 10×Gly was placed between 3×HA, 3×FLAG, or 3×Strep-tag II and the target protein, while a linker sequence GGSGGGGGG was placed between the target protein and 18×His (Fig. 1).

### Expression of fusion proteins with affinity tags

To validate these affinity tags in *Streptomyces*, an extracytoplasmic functions (ECF) sigma factor SigT from *S. coelicolor* was expressed from all above vectors. SigT can be physically protected by its cognate anti-sigma factor RstA from degradation, and is involved in dual positive feedback regulation of morphogenesis and secondary metabolism of *S. coelicolor*. We have previously tagged it with 3×FLAG and eGFP for immuno-detection and immunoprecipitation^22, 23^. However, a 10-amino acid flexible linker rich in Pro and Gly was essential for the proper expression of SigT-GFP^22^, suggesting that GFP, which is 236-amino acid long, might interfere with the proper conformation of SigT.

**Figure 2:**
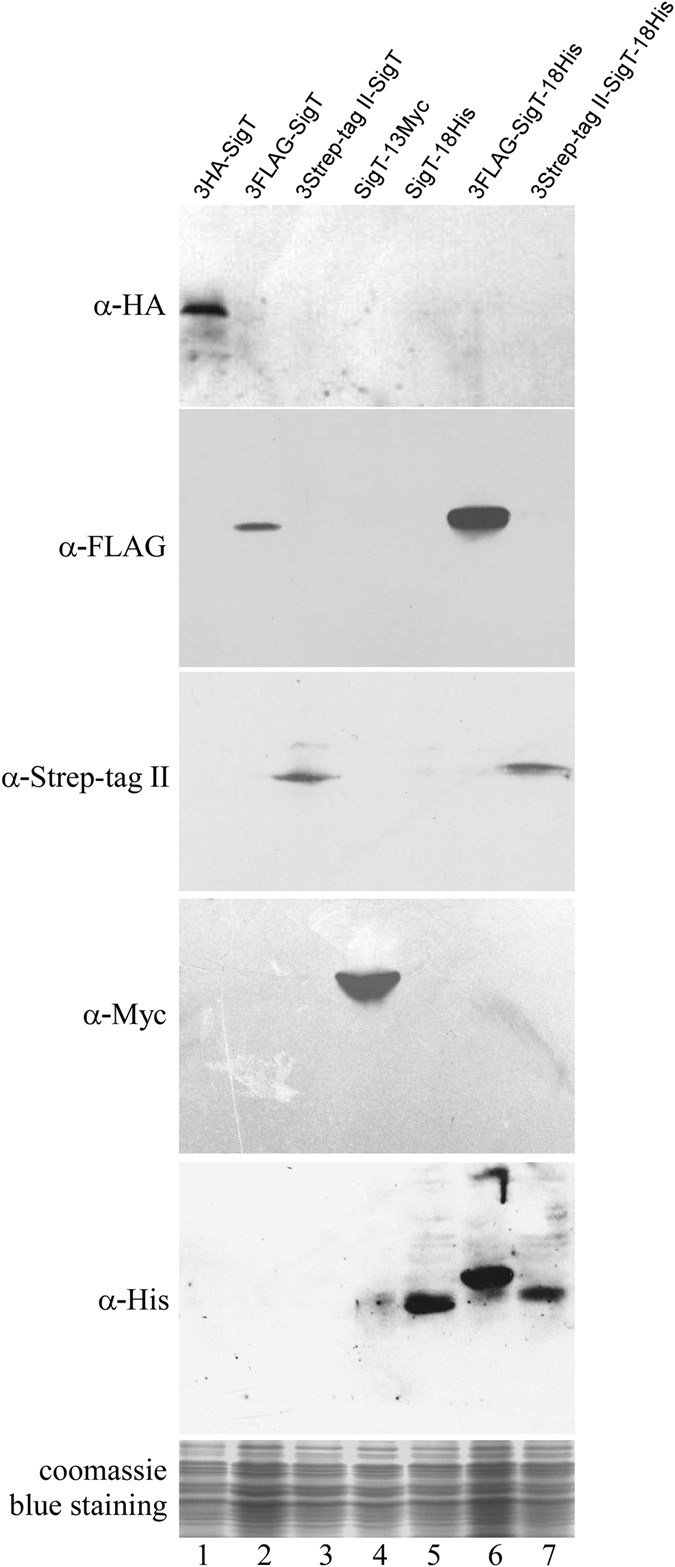
Western blot analysis of expression of SigT fused with series affinity tags in *S. coelicolor* M145 from pSN2-pSN8. All samples were immuno-blotted with tag-specific antibodies, and coomassie blue staining of total protein served as a loading control.

*sigT* coding sequence was cloned in all constructs in fusion with tags, and expressed in *S. coelicolor*. Immuno-blot assays showed SigT fusion proteins were successfully expressed in *Streptomyces* with α-FLAG, HA, Myc, His and Strep-tag II antibodies, respectively (Fig. 2). All the antibodies specifically recognized the corresponding epitopes, since only the SigT fusion protein bands were clearly observed in the immuno-blots. This highly reduced cross-reactivity will guarantee the exclusion of the false positive in downstream assays, especially such as ChIP. All the data suggested that these tags could be readily expressed and fused with *Streptomyces* proteins.

### Identification of SigT-interactive proteins by tandem affinity purification (TAP)

Previous report has shown that an artificial extension of target proteins with 3×FLAG tag will not disturb the functionality *in vivo* by ChIP assays in studying protein-DNA interaction^20^. Here we further validated the feasibility of these affinity tags in the tandem affinity purification (TAP), which is a powerful tool in exploring the protein-protein interaction *in vivo* ^36^.

**Figure 3:**
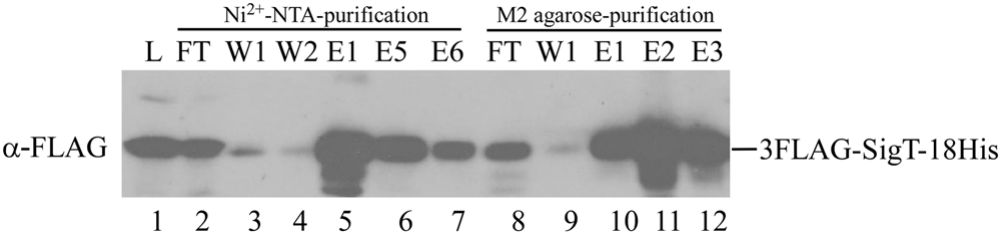
Immuno-blot assays of samples during tandem affinity purification (TAP) with anti-FLAG antibody. Total protein from *S. coelicolor ΔprcB/A* expressing 3FLAG-SigT-18His was subject to sequential nickel-based and anti-FLAG antibody-based affinity purification. Samples were taken during TAP as described in Materials and Methods. L: total lysate. FT: flow through. W: wash. E: elution.

SigT has been shown subject to protein degradation during the secondary metabolism of *S. coelicolor* ^23^, but its degradation was blocked in the proteasome-deficient *ΔprcB/A* mutant^21^, suggesting that SigT remain intact while interacting with other proteins involved in its degradation in this mutant. Therefore the TAP assay was demonstrated with SigT as a model in the *ΔprcB/A* mutant to explore its potential interactome (Fig. 3). SigT was expressed in pSN7 with dual tags 3×FLAG at its N-terminus and 18×His at C-terminus. 3FLAG-SigT-18His could be immuno-detected in the *ΔprcB/A* mutant (Fig. 3, lane 1). Though some SigT fusion protein flowed away after loading on Ni^2+^-NTA, initial affinity binding to nickel could capture most SigT protein (Fig. 3, lane 2–7). Then the eluent was reloaded on the anti-FLAG M2 monoclonal antibody-conjugated agarose. After careful washes to a low background, SigT together with its interactive-proteins dissociated from the beads by heat denature (Fig. 3, lane 8–12). After buffer exchange and trypsin digestion, the final eluent was subject to HPLC-MS/MS for peptide sequencing and identification.

Totally 28 proteins were identified in this TAP assay (Table 1) (see Supplementary data for the detailed information). Among them, SigT ranked No.2 with 100% group probability and showed 3 independent sequenced peptides, consistent with this protein as a bait in TAP. Interestingly, the anti-sigma factor RstA (SCO3891) had the most total independent spectra, and similarly, three peptides were independently sequenced as SigT, while all other 26 proteins only had one matched peptide. These data were consistent with our previous immunoprecipitation assay that SigT could physically interact with RstA^22^. Moreover, other proteins potentially involved in biosynthesis, regulation, transportation, etc, together with several proteins with unknown functions were also identified, suggesting that SigT might participate in diverse physiological processes by protein-protein interaction. All these data suggested that our dual tag strategy could be applied in TAP to study protein-protein interaction in *Streptomyces*.

**Table 1:** Protein identification by HPLC-MS/MS after tandem affinity purification (TAP).

Entry No.	Group probability	Total independent spectra	Protein description
1	1	19	SCO3891, anti-sigma factor RstA
2	1	14	SCO3892, RNA polymerase sigma factor SigT
3	1	3	SCO7274, hypothetical protein
4	0.9984	1	SCO0826, hypothetical protein
5	0.9984	1	SCO0908, hypothetical protein
6	0.9984	1	SCO5910, hypothetical protein
7	0.968	1	SCO0732, protease
8	0.9666	1	SCO4667, two-component system sensor kinase
9	0.9591	1	SCO0457, hypothetical protein
10	0.9538	1	SCO6950, hypothetical protein
11	0.9481	1	SCO0841, oxidoreductase
12	0.9453	1	SCO1218, transmembrane transport protein
13	0.9149	1	SCO3902, hypothetical protein
14	0.9107	1	SCO2295, hypothetical protein
15	0.9009	1	SCO5443, alpha-amylase
16	0.8982	1	SCO2086, UDP-N-acetylmuramoyl-L-alanyl-D-glutamate synthetase, MurD
17	0.8935	1	SCO2935, transcriptional regulator
18	0.8911	1	SCO1849, cobalamin biosynthesis protein, or cobaltochelatase subunit CobN
19	0.8785	1	SCO3857, regulatory protein
20	0.8738	1	SCO5486, pyridoxal-phosphate-dependent aminotransferase
21	0.8699	1	SCO3980, hypothetical protein
22	0.8641	1	SCO1813, GntR family transcriptional regulator
23	0.8606	1	SCO6849, hypothetical protein
23	0.8606	1	SCO6849, hypothetical protein
24	0.8329	1	SCO5883, hypothetical protein
25	0.8265	1	SCO3014, translation initiation factor
26	0.8198	1	SCO6334, transcriptional regulator
27	0.8152	5	SCO2015, nucleotidase
28	0.8152	2	SCO4581, hypothetical protein

## Discussion

The filamentous bacteria *Streptomyces* are becoming the focus for its industrial values to produce numerous secondary metabolites in medicinal and agricultural applications. High through-put genome sequencing also showed a large biosynthetic gene cluster pool in this genus, suggesting a huge capacity for natural product production. Many genetic tools and *in vitro* assays have been established to reveal the genetic circuits and pathway cross-talks controlling the physiology of *Streptomyces*. Here we provided biochemical approaches with several affinity tags for *in vivo* protein expression and detection, and also for the first time established tandem affinity purification (TAP) in *Streptomyces* to study protein-protein interaction with SigT as a bait. These *in vivo* biochemical tools can also be expanded to explore the real-time dynamics of protein-DNA interaction (such as ChIP), to establish the protein-protein interaction network (or interactome), and to examine protein-protein competition or coordination in regulating gene expression on the promoters of regulons, etc. All these mechanisms in *Streptomyces* will be the basis of building up biological models of their morphological development and rational designing of biosynthetic and regulatory circuits to exploit their potentialities in secondary metabolite production to the utmost.

## Materials and Methods

### Strains and media

*Streptomyces coelicolor* wild type M145 and the *ΔprcB/A* mutant^21^ were used for protein expression *in vivo*. *Escherichia coli* strain TG1 was a host for the routine plasmid sub-cloning. *E. coli* strain ET12456 containing pUZ8002 was used for conjugation of plasmids from *E. coli* to *Streptomyces* ^8^.

All *E. coli* cells were cultured in LB medium. The inter-species conjugations were demonstrated according to the protocol described on the SFM medium^8^. For SigT fusion protein expression, all *Streptomyces* strains were cultured in TSB supplemented with 5% PEG6000 (TSBP) to the logarithmic stage. For the TAP assay, the *S. coelicolor ΔprcB/A* mutant expressing 3FLAG-SigT-18His was initially cultured in TSBP for 1 day, and then transferred to the liquid R5- medium for the secondary metabolism development after continuous culture for 2 days^21^.

### Plasmid construction

All the plasmids and primers in this study were listed in Supplementary data Tables S1 and S2, respectively. Fragments I, II, III and IV containing *Nde*I-3HA-linker-MCS-TAA-*Not*I, *Nde*I-3FLAG-linker-MCS-linker-18His-TGA-*Not*I, *Nde*I-3Strep-tag II-linker-MCS-TAA-*Not*I and *Nde*I-MCS-13Myc-TAA-*Not*I (herein TAA and TGA encode the stop codons, and MCS is multiple cloning sites), respectively, were synthesized with optimized-codons for *Streptomyces* in Qinglan company, China (see Supplementary data for detailed sequences). ermEp* was amplified with primers 1 and 2 from pIJ8630-ermEp*^22^, digested with *Bam*HI, and inserted into the *Bgl*II site of pIJ8660^35^ to create the vector pIJ8660-ermEp*. Then the *egfp* gene was replaced with fragments I-IV at the *Nde*I/*Not*I site to give rise to plasmids pSN2, 7, 4 and 5, respectively. *Bgl*II/*Eco*RI fragments from pSN2 and pSN7 were cloned into *Bgl*II/*Eco*RI-digested pSN5 to create plasmids pSN1 and pSN6, respectively. Then pSN1 was digested by *Bgl*II and *Eco*RI, and the smaller DNA fragment was gel-recovered and inserted into *Bgl*II/*Eco*RI-digested pSN7 to make plasmid pSN3. *Bgl*II/*Eco*RI fragment of pSN4 were replaced with *Bgl*II/*Eco*RI fragment from pSN7 to produce plasmid pSN8. *sigT* was amplified from the genomic DNA of M145 with primers 3 and 4, digested with *Bgl*II and *Xba*I, and ligated into the *Bgl*II/*Xba*I site of pSN2 to pSN8 to create *sigT* expression plasmids as listed in Table S1.

### Western blot

*Streptomyces* total protein was prepared from the mycelia and Western blot was demonstrated as described previously with antibodies against HA (Tiangen, China), Myc (Tiangen, China), FLAG (Sigma, USA), His (Abmart), Strep-tag II (IBA, Germany)^21^. The primary antibodies were diluted in 1:2000, while the anti-mouse HRP-conjugated secondary antibody was diluted in 1:5000.

### Tandem affinity purification (TAP) and protein identification

The total protein was prepared from mycelia of the *ΔprcB/A* mutant expressing 3FLAG-SigT-18His in buffer A (50 mM Tris-HCl pH 8.0, 100 mM NaCl, 10 mM imidazole). Initial nickel-based purification was described by the manufacture (Merck). Briefly, the lysate was loaded on the buffer A-equilibrated Ni^2+^-NTA (the flow-through as FT), and incubated for 2 hours at 4 °C. After two rounds of washing with 10-column volumes of buffer A (W1, W2), the binding proteins were eluted with buffer B (50 mM Tris-HCl pH 8.0, 100 mM NaCl, 5% glycerol, 500 mM imidazole) for 6 times, each with 500 μl of buffer B (E1-E6). All the eluents were combined and EDTA was added to a final concentration of 1 mM. Then the protein mixture was incubated with 250 μl of anti-FLAG M2 agarose gel (Sigma) equilibrated with buffer C (50 mM Tris-HCl, pH 7.5, 150 mM NaCl) for 2 hours at 4 °C. After a short centrifugation (500 rpm, 3 min), the supernatant (FT) was discarded and the pellet was washed 3 times with 1 ml of buffer C (W1). After centrifugation at 500 rpm for 3 min, the supernatant was discarded as much as possible. 500 μl of 20 mM Tris-HCl (pH 8.0) buffer was added to the pellet and boiled for 5 min for protein elution, and this process was repeated for 2 times (E1-E3). All the eluents were combined and the purified proteins were digested with trypsin and identified by HPLC/MS/MS as described before^37^.

## Electronic supplementary material

Supplementary data
