# Supplementary material for: Development of Series of Affinity Tags in Streptomyces
Source: Sci Rep. 2017 Jul 31;7:6854. doi: 10.1038/s41598-017-07377-4 (PMC5537264; doi:10.1038/s41598-017-07377-4)
Supplement: Supplementary file 1 — Supplementary data [file 41598_2017_7377_MOESM1_ESM.pdf]

Development of Series of Affinity Tags in *Streptomyces*

Xu-Ming Mao*[1,2], Ning Sun*#[1,2], Yang Zheng[1,2], Yong-Quan Li¶[1,2]

[1]Institute of Pharmaceutical Biotechnology, College of Pharmaceutical Sciences, Zhejiang University, Hangzhou 310058, China

[2]Zhejiang Provincial Key Laboratory for Microbial Biochemistry and Metabolic Engineering, Hangzhou 310058, China

*These two authors contributed equally to this work.

¶To whom correspondence may be addressed:

Yong-Quan Li: College of Pharmaceutical Sciences, Zhejiang University, Hangzhou 310058, China. Tel: 86-571-88206632, Fax: 86-571-88208569. E-mail: lyq@zju.edu.cn.

Table S1: plasmids used in this study.

Name	Description	Reference
pIJ8630-ermEp*	*ermEp** in *Bam*HI site of pIJ8630	[1]
pIJ8660	Promoter-probing vector	[2]
pIJ8630-ermEp*	*ermEp** in *Bgl*II site of pIJ8660	This study
pSN1	*egfp* is replaced with MCS in pIJ8630-ermEp*	This study
pSN2	Affinity tag 3×HA at N-terminus	This study
pSN3	Affinity tag 3×FLAG at N-terminus	This study
pSN4	Affinity tag 3×Strep-tag II at N-terminus	This study
pSN5	Affinity tag 13×Myc at C-terminus	This study
pSN6	Affinity tag 18×His at C-terminus	This study
pSN7	Affinity tags 3×FLAG at N-terminus and 18×His at C-terminus	This study
pSN8	Affinity tags 3×Strep-tag II at N-terminus and 18×His at C-terminus	This study
pSN2-sigT	*sigT* at *Bgl*II/*Xba*I site of pSN2	This study
pSN3-sigT	*sigT* at *Bgl*II/*Xba*I site of pSN3	This study
pSN4-sigT	*sigT* at *Bgl*II/*Xba*I site of pSN4	This study
pSN5-sigT	*sigT* at *Bgl*II/*Xba*I site of pSN5	This study
pSN6-sigT	*sigT* at *Bgl*II/*Xba*I site of pSN6	This study
pSN7-sigT	*sigT* at *Bgl*II/*Xba*I site of pSN7	This study
pSN8-sigT	*sigT* at *Bgl*II/*Xba*I site of pSN8	This study

Table S2: primers used in this study.

No.	Sequence	Description
1	TCCTAAGGATCCGGCGGCTTGCGCCCGATGCTAGTC	*ermEp** amplification, For
2	AGCAGCGGATCCTACCAACCGGCACGATTG	*ermEp** amplification, Rev
3	AGATCTATGGCGGGCGGCGCGAGTCATG	*sigT* amplification, For
4	TCTAGACGCTCGTCCACCTCCGCCCTTC	*sigT* amplification, Rev

DNA sequence of the synthesized fragments I-IV:

>Fragment I: *Nde*I-3HA-linker-MCS-TAA-*Not*I

CATATGTACCCGTACGACGTCCCGGACTACGCCTACCCGTACGACGTCCCGGACTACGCCTACCCGTACGACG

TCCCGGACTACGCCGGCGGCGGCGGGGGCGGCGGCGGGGGCGGCAGATCTTGGATATCACATCGATCTTC

TAGACCGGGTACCGGCGGATCCTAAGCGGCCGC

>Fragment II: *Nde*I-3FLAG-linker-MCS-linker-18His-TGA-*Not*I

CATATGGACTACAAGGACCACGACGGCGATTACAAGGACCACGACATCGACTACAAGGACGACGACGACA

AGGGCGGCGGCGGCGGCGGCGGCGGCGGCGGCAGATCTTGGATATCACATCGATCTTCTAGACCGGGTAC

CGGCGGATCCGGCGGCGGCGGGGGCGGCCACCACCACCACCACCACCACCACCACCACCACCACCAC

CACCACCACCACTGAGCGGCCGC

>Fragment III: *Nde*I-3Strep tag-II-linker-MCS-TAA-*Not*I

CATATGGCCTCCTGGAGCCACCCGCAGTTCGAGAAGGGCGGCGGCTCGGGCGGGGGCAGCGGCGGCGGC

TCCTGGTCCCACCCCCAGTTCGAGAAGGGCGGCGGGAGCGGCGGCGGCTCCGGCGGCGGCAGCTGGTCG

CACCCGCAGTTCGAGAAGGGCGCCGGCGGCGGCGGGGGCGGCGGCGGGGGCGGCTCCAGATCTTGGAT

ATCACATCGATCTTCTAGACCGGGTACCGGCGGATCCTAAGCGGCCGC

>Fragment IV: *Nde*I-MCS-13Myc-TAA-*Not*I

CATATGCTGGGCAGATCTTGGATATCACATCGATCTTCTAGACCGGGTACCGGCGGATCCGAGCAGAAGCTG

ATCTCCGAGGAGGACCTGGAGCAGAAGCTGATCTCCGAGGAGGACCTGGAGCAGAAGCTGATCTCCGAG

GAGGACCTGGAGCAGAAGCTGATCTCCGAGGAGGACCTGGAGCAGAAGCTGATCTCCGAGGAGGACCTG

GAGCAGAAGCTGATCTCCGAGGAGGACCTGGAGCAGAAGCTGATCTCCGAGGAGGACCTGGAGCAGAAG

CTGATCTCCGAGGAGGACCTGGAGCAGAAGCTGATCTCCGAGGAGGACCTGGAGCAGAAGCTGATCTCCG

AGGAGGACCTGGAGCAGAAGCTGATCTCCGAGGAGGACCTGGAGCAGAAGCTGATCTCCGAGGAGGACC

TGGAGCAGAAGCTGATCTCCGAGGAGGACCTGTAAGCGGCCGC

References:

1. Mao, X.M. et al. Involvement of SigT and RstA in the differentiation of *Streptomyces coelicolor*. *FEBS Lett* **583**, 3145-50 (2009).

2. Sun, J., Kelemen, G.H., Fernandez-Abalos, J.M. & Bibb, M.J. Green fluorescent protein as a reporter for spatial and temporal gene expression in *Streptomyces coelicolor* A3(2). *Microbiology* **145 (Pt 9)**, 2221-7 (1999).
